# Geographical and Varietal Traceability of Chinese Jujubes Based on Physical and Nutritional Characteristics

**DOI:** 10.3390/foods10102270

**Published:** 2021-09-25

**Authors:** Linxia Wu, Ling Li, Guoguang Zhang, Nan Jiang, Xihui Ouyang, Meng Wang

**Affiliations:** 1 Institute of Quality Standard and Testing Technology, BAAFS, No. 9 Middle Road of Shuguanghuayuan, Haidian District, Beijing 100097, China; wulx@brcast.org.cn (L.W.); jiangnan_fx@163.com (N.J.); 2 Beijing Center of AGRI-Products Quality and Safety, No. 6 Middle Road of Yumin, Xicheng District, Beijing 100029, China; bjhjfp@163.com (L.L.); wabj2458@sohu.com (G.Z.)

**Keywords:** Chinese jujubes, origin traceability, variety traceability, PCA, OPLS-DA

## Abstract

Chinese jujube fruits are known for their high nutritional and functional values. To protect advantageous regional jujube fruits, it is important to monitor quality indicators and trace the origin and variety. In this study, 31 quality indicators of Chinese jujubes collected from 6 main producing areas were determined. According to different origins and varieties, Chinese jujube fruits were divided into five and six categories, respectively. To simplify the parameters, eight of the main characteristics, namely, soluble sugar content, fresh mass, edible rate, Na, Mg, K, Zn, and cyclic adenosine monophosphate (cAMP), were screened based on multiple comparison, correlation analysis, and principal component analysis (PCA). According to the eight main parameters, it was found that that both the categorical and cross-validated classification accuracy of linear discriminant analysis (LDA) were 100%. The discrimination accuracy of the testing set samples based on the orthogonal partial least squares-discriminant analysis (OPLS-DA) model were 90 and 93% for geographical and varietal classification, respectively. This indicated that the eight main parameters could be used as the characteristic parameters for the origin and variety traceability of Chinese jujubes.

## 1. Introduction

Jujube (*Ziziphusjujuba Mill*.) has a long history of cultivation, and it is widely distributed across Asian countries, including China, and in southeastern Europe, particularly in Spain, Italy, and Malta [1]. Jujube is famous for its high nutritional and functional value, because it is rich in soluble sugar, trace elements, phenolics, organic acids, triterpene acids, and cyclic adenosine monophosphate (cAMP) [2,3]. Therein, phenolics and triterpene acids have been discovered to have many health benefits including antioxidant activity and anticarcinogenic properties [4]. cAMP is a derivative of nucleotide, which has the function of being a secondary messenger and participates in the regulation of a wide range of physiological and biochemical processes [5].

Therefore, a number of national, provincial, ministerial and regional level programs applied to jujube fruits [1,6]. China has the highest output of red jujubes in the world—6.873 milliontons [4]. More than 90% of fresh jujubes in China’s total output are dried to prevent microbial reproduction and prolong the storage time [7]. In China, jujube is widely consumed as food and is used in traditional Chinese medicine. It has antitumor properties, improves the cardiovascular and cerebrovascular systems, and enhances human immunity and hematopoietic function [8]. The physical and chemical properties of jujubes are affected by their origin, because the edaphoclimatic conditions vary between regions [9].

In recent years, food quality and security issues, such as the variety of raw materials, batch mixing, and resource transformation, have occurred frequently, which are related to the livelihood of people, social stability, and healthy development of the national economy [10]. To avoid food quality and safety problems, countries all over the world require strict tracing and monitoring of the whole food production process ‘from farm to table’. Because it is cost-effective, time saving and universal, multiple mineral element analysis is one of the techniques mainly applied in traceability and adulteration studies. Gonzalez-Dominguez et al. [11] determined the content of sensory and health characteristics of strawberry, including sugars, organic acids, phenolic compounds, and essential and non-essential mineral elements, and the results showed a good identification result for cultivar and cultivation system differentiation. Zhang et al. [12] analyzed the nutrients and mineral elements of Tibet highland barley (*Hordeum vulgare* L.) and classified barley sources effectively by linear discriminant analysis. Sun et al. [13] identified the geographical origin of Chinese *Angelica* based on specific metal element fingerprinting. It demonstrated the feasibility of origin and variety traceability by nutritional characteristics. In order to improve the accuracy of such tracing, several simple physical parametersare combined with nutritional parameters in this study.

To protect advantageous regional Chinese jujubes, providing optimum benefits as functional foods, as well as tracing and verifying Chinese jujubes, the physical and nutritional characteristics of Chinese jujubes from different origins and varieties were studied. Based on principal component analysis (PCA), the main parameters for category differentiation were screened, which would objectively and comprehensively evaluate the internal quality of Chinese jujubes. Combined with the discriminant model, the origin and variety of Chinese jujubes were identified. This provided a theoretical basis for the main quality parameters’ determination of Chinese jujubes from different areas and varieties, which would be of great significance in the origin and variety traceability and production of Chinese jujubes.

## 2. Materials and Methods

### 2.1. Sampling Information

**Figure 1:**
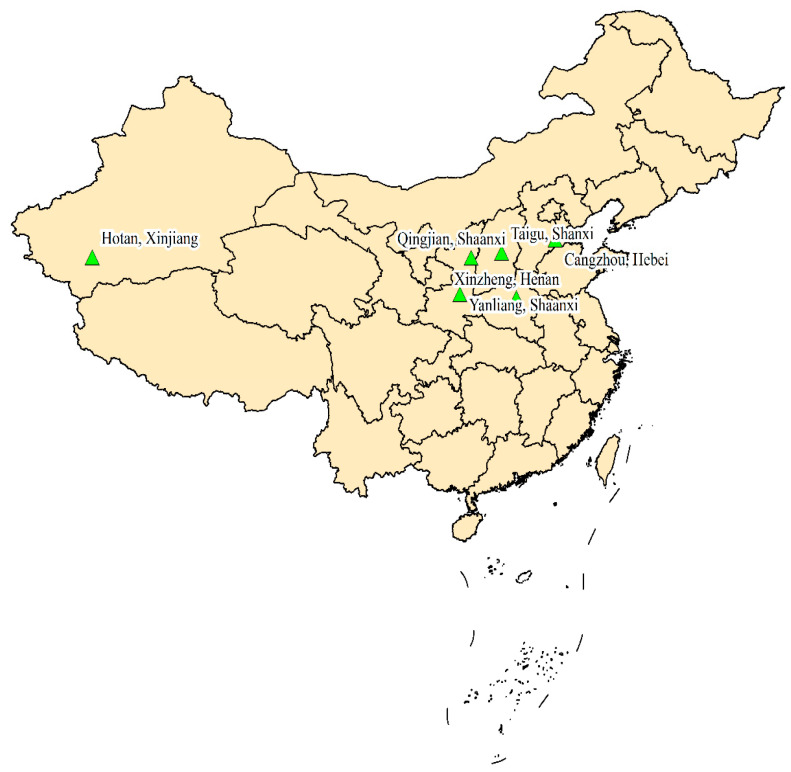
Sampling sites of Chinese jujube samples.

A total of 111 Chinese jujube samples were collected from five regions of China: Xinzheng (city of Henan), Cangzhou (city of Hebei), Taigu (county of Shanxi), Yanliang (district of Shaanxi), and Hotan (district of Xinjiang) (Figure 1). Six varieties were sampled, and each collection contained 12–21 samples (Table 1). Five replicates had been performed for each investigated sample. After the seed was removed, the resultant fruits were immediately homogenized. According to the differences in origin and variety, Chinese jujube samples were divided into five and six categories, respectively.

### 2.2. Quality Parameter Determination

#### 2.2.1. Fresh Fruit Mass Determination

The average fresh mass of jujube fruit was determined according to Chinese standard GB/T5835-2009. Briefly, 1000 g of Chinese jujube fruit from each variety from different origins was randomly selected and measured with an electronic balance. The average fresh mass of the fruit was calculated by dividing the total weight of the fruit by the fruit number. Each measurement was made in triplicate.

#### 2.2.2. Determination of Shape Ratio of Fruits

The shape ratio of jujube fruit refers to the ratio of polar diameter to equatorial diameter of the fruit [6]. By measuring the horizontal (equatorial diameter) and vertical distance (polar diameter) of the fruit (30 per category) using a digital caliper, the shape ratio was determined. Each measurement was conducted in triplicate.

#### 2.2.3. Edible Fruit Rate Determination

The edible rate of the jujube fruit was determined according to Chinese standard GB/T5835-2009. In this study, 200–300 g of healthy jujube fruit was measured. The flesh and stone were separated, and the flesh was weighed. The edible rate of the fruit was calculated by the flesh weight divided by the total weight of the fruit. Each measurement was performed in triplicate.

#### 2.2.4. Moisture Content Determination

According to GB 5009.3-2016, the moisture content of the Chinese jujubes was determined by drying 5 g samples in an air oven at a temperature of 105 °C until a constant weight was reached.

#### 2.2.5. Soluble Sugar Determination

Soluble sugar content was determined according to the Chinese standard NY/T2742-2015 by 3,5-dinitrosalicylic acid colorimetry. Zinc acetate solution was prepared by dissolving 21.6 g zinc acetate in a little water, adding 3 mL glacial acetic acid, and fixing the volume to 100 mL with water. Potassium ferrocyanide solution was prepared by dissolving 10.6 g potassium ferrocyanide in 100 mL water. Fruit sample homogenate (10.00 g) was transferred to a 250 mL volumetric flask. Zinc acetate solution (3.0 mL) and potassium ferrocyanide solution (3.0 mL) were slowly added, and then water was added to the scale. After standing for 2 min, the sample was filtered through filter paper. Aliquots of 0, 0.2, 0.4, …, 1.2 mL of standard solutions containing 1 mg/mL glucose were transferred to seven separate 10 mL glass tubes and diluted to 2.0 mL with water. Each solution was then mixed with 4.0 mL 3,5-dinitrosalicylic acid reagent. The absorbance of each solution was then measured in a 1.0 cm quartz cuvette at 540 nm after being heated for 5 min at 100 °C. A standard glucose absorbance curve was then obtained from these measurements. A mixture of 5.0 mL of each jujube fruit sample solution and 1.0 mL hydrochloric acid solution (6.0 mol/L) was heated for 10 min at 80 °C. After being cooled to room temperature, 3 drops of the methyl red parameter were added and neutralized with 6 mol/L NaOH solution to a light pink color. It was diluted to a volume of 100 mL and then measured as described above for the standard glucose solutions.

#### 2.2.6. Mineral Element Determination

Sodium, magnesium, potassium, manganese, iron, copper, and zinc were determined using inductively coupled plasma mass spectrometry after digestion in nitric acid according to Chinese standard GB5009.268-2016. Briefly, 0.2~0.5 g of solid sample (accurate to 0.001 g) was weighed or 1.00~3.00 mL of liquid sample was transferred into the microwave digestion inner tank. Firstly, we heated the sample containing ethanol or carbon dioxide at low temperature on the electric heating plate to remove ethanol or carbon dioxide, add 5~10 mL nitric acid, covered it and left it for 1 h or overnight, and tightened the tank cover. Digestion was carried out according to the standard operating steps of microwave digestion instrument (see Table S1 for digestion reference conditions). The digestion tank was taken out after cooling and put on the electric heating plate at 100 °C for 30 min or in an ultrasonic water bath for 2~5 min. Fixed the volume with water to 25~50 mL and mixed it well.

#### 2.2.7. Ascorbic Acid Determination

The ascorbic acid content was measured by high-performance liquid chromatography (HPLC) using the reference of Chinese standard GB5009.86-2016. The homogenized sample (0.5 g) was transferred to a 50 mL volumetric flask with 20 g/L partial phosphoric acid solution. It was shaken well and transferred to a 50 mL centrifuge tube. After ultrasonic extraction for 5 min, it was centrifuged for 5 min at 4000 r/min, and the supernatant was passed through a 0.45 μm water phase filter membrane. Finally, the filtrate was used for ascorbic acid analysis.

#### 2.2.8. Dietary Fiber Determination

The content of dietary fiber was analyzed according to Chinese standard GB5009.88-2014. After being desugared, 1.0 g samples were enzymatically digested with 50 mL α-amylase at a constant temperature of 95–100 °C for 35 min. According to the ratio of ethanol to sample solution volume of 4:1 in each sample enzymatic solution, 95% ethanol was added and precipitated at room temperature for 1 h. The residue was successively washed twice with 15 mL 78% ethanol, 15 mL 95% ethanol, and 15 mL acetone. After washing, the solution was removed by suction filtration, the residue was dried overnight at 105 °C, and total dietary fiber content was obtained.

#### 2.2.9. cAMP Determination

cAMP was analyzed according to a published method [14] with a minor modification. The sample (1.0 g) was transferred to a 50 mL volumetric flask. After adding 40 mL ultrapure water, it was extracted ultrasonically at 80 °C for 1 h. An appropriate amount of supernatant was filtered through a 0.45 μm membrane filter and injected into an HPLC with a 20 μL injection volume. An Agilent 1260 Infinity HPLC system equipped with an Agilent TC C18 column (4.6 mm × 250 mm, 5 μm) was used to separate cAMP. The mobile phase consisted of 20: 80 (*v/v*) methanol: 20 mmol/L KH_2_PO_4_ with a flow rate of 1 mL/min. The column was maintained at 30 °C during the elution program.

### 2.3. Statistical Analyses

Statistical data were analyzed using SPSS 22.0 (SPSS Inc., Armonk, NY, USA). Duncan’s multiple comparison test was performed to determine the significant differences between samples from different categories; a *p* < 0.05 value was considered to be significant. Correlation analysis (CA) was used to quantitatively analyze the relationship between the two variables. The raw data were converted to standardized data ranging between 0 and 1 before CA. Principal component analysis (PCA) was applied to reduce the dimension of the data and retain most of the information of the original data. The cumulative variance contribution rate usually reflected the ratio of the original information contained in the principal component (PC). After the data were rotated by the maximum variance method, the load value of each factor was closer to 0 or 1, which could better explain and summarize the factors under each PC.

PCA, partial least squares-discriminant analysis (PLS-DA), and orthogonal partial least squares-discriminant analysis (OPLS-DA) were conducted using SIMCA 14.1, (UMetrics AB, Umeå, Sweden) to cluster and distinguish samples in different categories. The goodness of fit in discriminant analysis was measured by the statistical parameter of R^2^ [15]. A total of 23 Chinese jujube samples were used as training sets and 13 samples as testing sets to verify the accuracy of the established OPLS-DA model. The predictive ability was expressed as the percentage of correctly classified samples relative to the total dataset. LDA was used to evaluate whether samples from different categories could be classified by certain parameters.

## 3. Results

### 3.1. Differences in Chinese Jujube Samples from Different Categories

As shown in Table 1 and Table S2 (contents of 15 individual amino acids are shown in Table S2), there were significant differences among the qualities of Chinese jujubes of 7 categories (*p* < 0.05). As seen in Figure S1, the ascorbic acid content in Huizao from Henan were the highest (*p* < 0.05). In Jinsixiaozao from Hebei, fresh mass and cAMP were higher than those in other categories (*p* < 0.05). The edible rate and moisture content in Hupingzao from Shanxi were significantly higher than those in other categories (*p* < 0.05). Compared with the other categories, the tyrosine and arginine content in Tanzao samples from Shaanxi were higher (*p* < 0.05). Except for the above 7 parameters, the remaining 24 parameters were highest in samples from Xinjiang (*p* < 0.05). This indicates that the nutritional and functional characteristics of Chinese jujubes were affected by their origin.

The characteristics of 31 Chinese jujube quality parameters are shown in Table S3. Among all parameters, the coefficient of variation (CV) of Na was the largest, which was 134.93%, indicating a significant difference for Na among different categories. The CV values of ascorbic acid content followed, with a range of 19.00–357.00 mg/100 g. The edible rate of Chinese jujubes from different categories had the smallest CV (1.35%), indicating no significant difference for edible rate among different categories, and it could be excluded from further screening of the most important quality parameters.

### 3.2. CA of Quality Parameters

CA was conducted to quantify the relationship among different quality parameters of Chinese jujubes. According to Table 2 (individual amino acids were not provided), there were significant positive correlations between Fe and Cu (*p* < 0.05). Highly significant negative correlations were found between ascorbic acid and soluble sugar content, moisture content and soluble sugar content, and moisture content and cAMP (*p* < 0.01). Edible rate and dietary fiber, Mg and Cu, and K and Mn presented highly positive correlations (*p* < 0.01). In general, correlations exist to a certain extent between some parameters, indicating that several quality parameters overlapped. To improve the classification efficiency and accuracy, it was necessary to further categorize and simplify the relevant parameters.

### 3.3. PCA Analysis for Main Parameter Selection and Quality Evaluation

**Figure 2:**
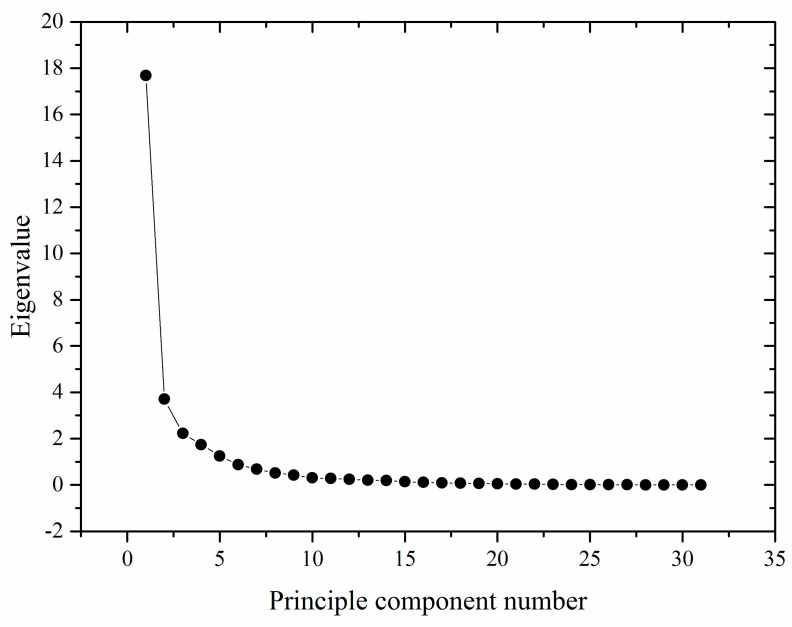
Contributions of the total variance accounted for by the PCs.

PCA was applied to decompose data into a few independent variables that could explain most of the original variance [16]. It has been widely adopted to simplify quality parameters and quality evaluations [16,17,18]. To evaluate the components accounting for most of the variability in raw data, the decomposition by PCA was graphically described in the form of a scree plot (Figure 2). It suggested that PCs with eigenvalues greater than 1 could be retained based on Kaiser’s rule [19]. In this study, the eigenvalues of the first five PCs were larger than 1, explaining 85.81% of the total variance. They accounted for 57.04, 11.95, 7.17, 5.62, and 4.03%, respectively, of the total variation in the data (Figure 1). The maximum variation of the dataset was represented by the first PC. To clarify the details of each PC represented, varimax rotation was performed to further analyze the quality parameter values. As a modification of coordinates used in PCA, varimax rotation maximized the sum of the variances of the squared loadings [20].

The maximum variance method is to find the rotational load that can maximize the variance of the square of the load in each column of the load matrix. After the data were rotated by the varimax method, the factor loadings were closer to 0 or 1, which could better explain and summarize the factors under each PC. The greater the factor loadings, the higher the contribution ratio under the PC. The results of PCA after rotation are shown in Table 3. K, Mg, soluble sugar content, and Na had a positive correlation with the first PC; however, the moisture content was negative. Because there were highly negative correlations between soluble sugar content and moisture content, moisture content had a lower loading value, excluding it from the main quality parameters. For PC2, phenylalanine, arginine, lysine, leucine, tyrosine, and histidine were the main influencing parameters. Therefore, no main quality parameter was selected from PC2. The main influencing parameters of PC3 were fresh mass, cAMP, and edible rate. Shape ratio and Zn were the main influencing parameters in the fourth and fifth PCs, respectively. However, the varimax rotated factor loadings of PC2 and PC4 were not high (<0.8); therefore, no main parameter was screened from them. As a result, soluble sugar content (X1), fresh mass (X3), edible rate (X5), Na (X7), Mg (X8), K (X9), Zn (X13), and cAMP (X15) were selected as the main parameters.

Dividing the PC loadings of each parameter by the square root of its corresponding eigenvalue, the weight coefficients of 31 quality parameters and the expressions of 5PCs were obtained [21]. By the standardized data and the weights of five characteristic quality factors, the synthesis scores and ranking of Chinese jujubes is presented in Table S4. The categories with a synthesis score from high to low were Xinjiang Junzao, Xinjiang Huizao, Shaanxi Tanzao, Shaanxi Xiangzao, Shanxi Hupingzao, Henan Huizao, and Hebei Jinsixiaozao. Xingjiang, Junzao, with the highest score, had the best comprehensive quality, followed by Xinjiang Huizao.

### 3.4. Chemometric Analysis

Other than reducing the dimensionality of numerical datasets in a multivariate problem, PCA can also classify samples into different groups as a classical unsupervised algorithm of pattern recognition [16]. PLS-DA and OPLS-DA were prevalently applied to discriminate two or more groups; multi-classification enabled the simultaneous modeling of multiple classes [22].

**Figure 3:**
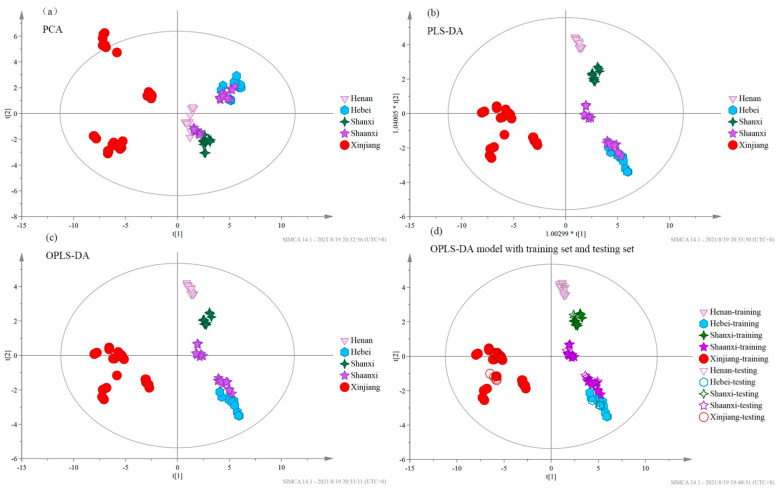
Principal component analysis (PCA), partial least squares-discriminant analysis (PLS-DA), and orthogonal partial least squares-discriminant analysis (OPLS-DA) of Chinese jujube samples in five regions with the eight main quality parameters, and the OPLS-DA model of the five categories of Chinese jujubes samples with the training set and testing set: (**a**) PCA; (**b**) PLS-DA; (**c**) OPLS-DA; (**d**) OPLS-DA model with training set and testing set.

To discriminate regionally different Chinese jujube samples, PCA, PLS-DA, and OPLS-DA analyses were conducted on the basis of the eight main quality parameters in Chinese jujubes (Figure 3a–c). The OPLS-DA (R^2^ = 0.912) model clearly classified samples into five different categories. In contrast, PCA (R^2^ = 0.81) and PLS-DA (R^2^ = 0.9) models had poor classification and discrimination. Linear discriminant analysis of the eight variables in Chinese jujubes showed that the correct classification rate of the original dataset and the accuracy of leave-one-out cross-validation was 100%. To test the accuracy of the model, 70% of the data was used as training set and 30% of the data was used as testing set. The testing set samples were divided into different categories by the OPLS-DA model (Figure 3d). Each column of the confusion matrix represents the prediction category, and each row represents the true category (Table 4(a)). The discrimination accuracy of samples in five categories was 100% in the training set and 71.43–100% in the testing set.

**Figure 4:**
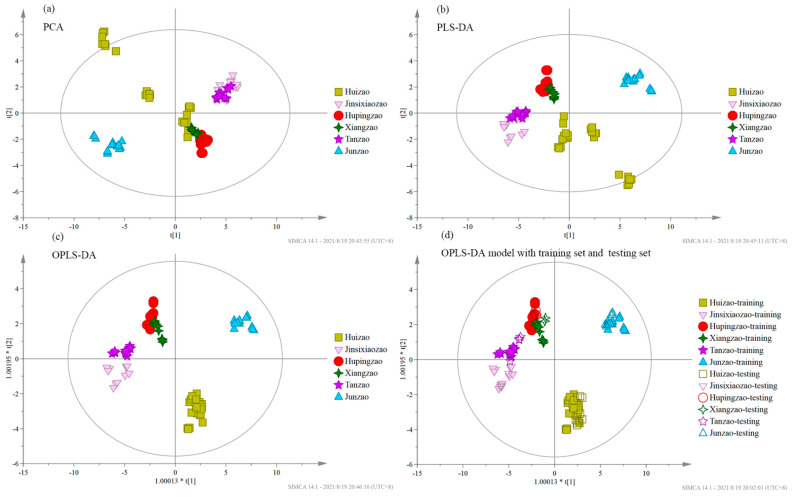
Principal component analysis (PCA), partial least squares-discriminant analysis (PLS-DA), and orthogonal partial least squares-discriminant analysis (OPLS-DA) of Chinese jujube samples in six varieties with the eight main quality parameters, and the OPLS-DA model of the six categories of Chinese jujubes samples with the training set and testing set: (**a**) PCA; (**b**) PLS-DA; (**c**) OPLS-DA; (**d**) OPLS-DA model with training set and testing set.

For varietally different Chinese jujube samples discrimination, the OPLS-DA (R^2^ = 0.924) model clearly classified samples into six different categories. In contrast, PCA (R^2^ = 0.905) and PLS-DA (R^2^ = 0.916) models had poor classification and discrimination (Figure 4a–c). Linear discriminant analysis of the eight variables in Chinese jujubes showed that the correct classification rate of the original dataset and the accuracy of cross-validation was 100%. To test the accuracy of the model, the testing set samples were divided into different categories by the OPLS-DA model (Figure 4d). The discrimination accuracy of the samples in six categories was 100% in the training set and 75–100% in the testing set (Table 4(b)).

## 4. Discussion

Chinese jujubes have a high nutritional value, and they are especially rich in ascorbic acid and cAMP. For ascorbic acid, the content is similar to that of kiwifruit [23] and 30 times more than that in cherry [24]. The lower concentrations of ascorbic acid in the Xinjiang region may be because it was lost during the special drying process with fruit air drying on the tree. As the second messenger in the organism’s cells, the level of cAMP in mature jujube was 30–160 μg/g, the highest amount which was observed in more than 180 natural plants [25]. In this study, cAMP levels in Xinjiang Junzao reached up to 213.29 mg/kg. Chinese jujube is an important source of K, Zn, Mg, Na, and Fe [26]. The high K and low Na contents in Chinese jujubes are good for people with hypertension problems [27,28]. Chinese jujubes can be used as a supplement for Fe deficiency without any side effects, such as nausea, headaches, and anorexia, which may occur with iron tablet supplements [29].

The nutritional value of Chinese jujubes differs with origin and variety. Generally, Xinjiang Junzao and Huizao had the best comprehensive quality, which may be related to the geographical environment. Although genotype is the main factor determining fruit physical and chemical properties, geographical environment and management techniques affect fruit nutrient biosynthesis and metabolism [30]. Being located in the northwest of China, Xinjiang possesses a high day-to-night temperature difference, long sunshine duration, high sunshine intensity, and a low rainfall amount. It benefits the accumulation of nutrients in jujube fruits, resulting in high quality jujube fruits in the Xinjiang region [6].

There were correlations to a certain extent among quality parameters. The positive correlation between edible rate and dietary fiber that was observed in this study had also been reported by Bi et al. [20]. Gao et al. [31] also found that moisture content and soluble sugar content had significant negative correlations. Highly significant negative correlations between moisture content and cAMP were consistent with Chen et al. [6]. However, there was a lack of literature on other significant correlations. More studies should be conducted to validate the correlation between different indices and explore the mechanisms behind these correlations.

Different methods were effectively used in the traceability of the origin and variety of Chinese jujubes. PCA and LDA were conducted to classify and differentiate 21 jujube cultivars using 24 fatty acids. Most groups were clearly separated [32]. The fatty acid methyl ester profiles of fruit peels and pulp of *Ziziphus jujuba* could be used to discriminate four Spanish cultivars with the LDA model [33]. By machine vision, a multilayer perceptron neural network could be used to classify jujube fruits into four qualitative grades with an accuracy of 98.61% [34]. Near-infrared spectroscopy was applied for the geographical origin classification of jujube fruit samples. PCA provided a useful qualitative technique for the discrimination of jujube fruits [35]. In this study, the origin and variety traceability of Chinese jujubes was realized from the perspective of physical and nutritional characteristics. Compared with PCA and PLS-DA, the OPLS-DA classification model discriminated Chinese jujube samples with the highest prediction performance. OPLS is a variant of PLS in which the PLS model is rotated, placing the Y -predictive part of the model in the first component [36]. OPLS-DA had a stronger interpretation ability because it could filter out the signals irrelevant to the model by integrating an orthogonal signal correction filter with PLS.

## 5. Conclusions

In this study, samples of six varieties of Chinese jujube from five regions of China were collected. A total of 31 quality parameters based on physical and nutritional characteristics were determined. Duncan’s multiple comparison, CA, and PCA were successfully applied for screening characteristic quality parameters to discriminate Chinese jujubes of different categories. Duncan’s multiple comparison showed that the quality of Chinese jujubes of different categories was significantly different (*p* < 0.05). CA indicated that positive or negative correlations existed to a certain extent between some quality parameters. Through PCA, eight main quality parameters were finally obtained from five PCs: soluble sugar content (X1), fresh mass (X3), edible rate (X5), Na (X7), Mg (X8), K (X9), Zn (X13), and cAMP (X15). Based on the eight screened characteristic parameters, a strong discrimination result based on the geographical and varietal origin of Chinese jujubes was exerted by multivariate statistical analysis (PCA, PLS-DA, OPLS-DA). The source and variety of blind samples could also be correctly determined by the established discrimination model. Based on the OPLS-DA model, the discrimination accuracy of the testing samples was 90% and 93% for geographical and varietal classification, respectively. The original and cross-validated correct rates of LDA were 100%. By selecting the eight characteristic parameters from 31 parameters, it simplified the origin and variety traceability process and improved the efficiency, which might be applied in the food industry for quality control.

## Figures and Tables

**Table 1:** Determination of 16 physical and nutritional parameters in Chinese jujubes from different categories.

Quality Parameters		Henan Huizao	Hebei Jinsixiaozao	Shanxi Hupingzao	Shaanxi Xiangzao	Shaanxi Tanzao	Xinjiang Junzao	Xinjiang Huizao
Sample number		15	15	15	12	15	21	18
Physical parameters	Fresh mass (g)	10.41 ± 0.72 ^d^	7.50 ± 0.80 ^d^	18.08 ± 2.41 ^b^	14.63 ± 0.73 ^c^	10.26 ± 1.42 ^d^	25.96 ± 3.66 ^a^	7.84 ± 3.00 ^d^
	Shape ratio	1.45 ± 0.07 ^ab^	1.35 ± 0.02 ^bc^	1.28 ± 0.12 ^bc^	1.18 ± 0.02 ^c^	1.28 ± 0.32 ^bc^	1.57 ± 0.10 ^a^	1.40 ± 0.06 ^ab^
	Edible rate (%)	92.67 ± 0.22 ^c^	92.58 ± 0.70 ^c^	95.47 ± 0.30 ^a^	94.29 ± 0.21 ^b^	93.70 ± 1.13 ^b^	95.41 ± 0.99 ^a^	93.82 ± 0.49 ^b^
	Soluble sugar (%)	24.28 ± 1.16 ^c^	19.94 ± 1.88 ^d^	23.26 ± 1.08 ^c^	23.93 ± 1.00 ^c^	22.66 ± 2.58 ^c^	37.93 ± 0.72 ^b^	39.77 ± 0.61 ^a^
	Moisture content (%)	65.18 ± 2.71 ^a^	65.12 ± 0.80 ^a^	68.75 ± 2.45 ^a^	67.66 ± 0.72 ^a^	64.41 ± 0.72 ^a^	41.35 ± 0.45 ^b^	38.46 ± 6.59 ^b^
Nutritional parameters	Ascorbic acid (mg/100 g)	331.80 ± 1.88 ^a^	330.40 ± 18.08 ^a^	168.20 ± 30.33 ^b^	113.00 ± 16.73 ^c^	186.40 ± 10.67 ^b^	70.50 ± 2.74 ^d^	34.17 ± 13.64 ^e^
	Na (mg/kg)	3.78 ± 0.58 ^c^	56.04 ± 16.42 ^c^	27.00 ± 13.06 ^c^	24.38 ± 3.76 ^c^	9.12 ± 2.06 ^c^	149.95 ± 21.11 ^b^	357.05 ± 127.88 ^a^
	Mg (mg/kg)	243.80 ± 12.52 ^c^	208.60 ± 25.39 ^cd^	147.80 ± 10.57 ^f^	182.25 ± 11.15 ^de^	168.40 ± 5.77 ^de^	305.00 ± 35.89 ^b^	400.33 ± 75.58 ^a^
	K (mg/kg)	3652.20 ± 366.73 ^c^	2908.40 ± 220.97 ^c^	3024.40 ± 278.62 ^c^	3229.00 ± 298.67 ^c^	2066.40 ± 280.09 ^d^	6307.17 ± 637.33 ^b^	7183.67 ± 1156.97 ^a^
	Mn (mg/kg)	2.34 ± 0.19 ^ab^	1.70 ± 0.30 ^c^	2.08 ± 0.38 ^bc^	1.65 ± 0.10 ^c^	2.24 ± 0.50 ^ab^	2.67 ± 0.36 ^a^	2.67 ± 0.36 ^a^
	Fe (mg/kg)	5.10 ± 0.38 ^c^	4.50 ± 0.49 ^c^	4.22 ± 0.63 ^c^	3.60 ± 0.59 ^c^	7.64 ± 6.37 ^bc^	10.97 ± 1.68 ^ab^	14.57 ± 3.65 ^a^
	Cu (mg/kg)	1.39 ± 0.23 ^b^	1.23 ± 0.23 ^bc^	1.27 ± 0.12 ^bc^	1.25 ± 0.13 ^bc^	1.06 ± 0.21 ^bc^	0.88 ± 0.22 ^c^	1.81 ± 0.54 ^a^
	Zn (mg/kg)	2.46 ± 0.59 ^cd^	3.48 ± 0.47 ^a^	3.18 ± 0.64 ^ab^	1.98 ± 0.22 ^d^	3.34 ± 0.54 ^ab^	2.67 ± 0.72 ^bc^	3.30 ± 0.45 ^ab^
	dietary fiber (%)	4.54 ± 0.03 ^f^	6.23 ± 0.04 ^c^	6.07 ± 0.03 ^d^	5.94 ± 0.04 ^e^	6.10 ± 0.03 ^d^	6.64 ± 0.04 ^b^	6.76 ± 0.15 ^a^
	cAMP (mg/kg)	45.15 ± 20.05 ^c^	41.92 ± 11.29 ^c^	93.01 ± 13.95 ^b^	57.06 ± 21.07 ^c^	41.96 ± 4.59 ^c^	213.29 ± 34.14 ^a^	90.86 ± 12.99 ^b^
	Total amino acid (mg/100 g)	1.16 ± 0.10 ^cd^	1.04 ± 0.06 ^d^	1.08 ± 0.05 ^cd^	1.36 ± 0.04 ^b^	1.22 ± 0.03 ^bc^	1.81 ± 0.10 ^a^	1.82 ± 0.24 ^a^

Values are means ± SD. Numbers with different superscript are significantly (*p* < 0.05) different with respect to column for the different categories: Duncan’s multiple comparison test.

**Table 2:** Correlation analysis (CA) on quality parameters.

	X1	X2	X3	X4	X5	X6	X7	X8	X9	X10	X11	X12	X13	X14	X15	X31
X1	1															
X2	−0.777 **	1														
X3	0.319	−0.381	1													
X4	0.393	−0.067	0.217	1												
X5	0.351	−0.593	0.798	0.016	1											
X6	−0.945 **	0.698	−0.205	−0.496	−0.216	1										
X7	0.788	−0.624	−0.032	0.221	0.137	−0.742	1									
X8	0.836	−0.496	−0.049	0.444	−0.001	−0.882	0.734	1								
X9	0.928	−0.661	0.205	0.439	0.233	−0.934	0.745	0.931	1							
X10	0.622	−0.423	0.208	0.562	0.219	−0.666	0.422	0.666	0.657 **	1						
X11	0.783	−0.599	−0.014	0.366	0.08	−0.826	0.621	0.793	0.765	0.729	1					
X12	0.19	−0.111	−0.587	0.038	−0.395	−0.258	0.267	0.430 **	0.367	0.201	0.344 *	1				
X13	−0.008	0.06	−0.256	0.117	−0.085	−0.097	0.158	0.169	0.045	0.315	0.296	0.192	1			
X14	0.553	−0.717	0.23	0.071	0.425 **	−0.603	0.600	0.390	0.487	0.203	0.517	−0.039	0.313	1		
X15	0.678	−0.586	0.761	0.458	0.598	−0.654 **	0.315	0.38	0.627	0.409	0.408	−0.232	−0.201	0.483	1	
X31	0.913	−0.796	0.272	0.389	0.305	−0.926	0.627	0.805	0.894	0.569	0.766	0.222	−0.13	0.562	0.689	1

Note: ** and * mean significant level at 0.01 and 0.05, respectively. X1–X15, X31: soluble sugar (%), ascorbic acid (mg/100 g), fresh mass (g), shape ratio, edible rate (%), moisture content (%), Na (mg/kg), Mg (mg/kg), K (mg/kg), Mn (mg/kg), Fe (mg/kg), Cu (mg/kg), Zn (mg/kg), dietary fiber (%), cAMP (mg/kg), totalamino acid (mg/100 g).

**Table 3:** Varimax rotated factor loadings of the first five principal components (PCs).

Quality Parameter	PC1	PC2	PC3	PC4	PC5
Soluble sugar (X1, %)	0.859	0.366	0.180	0.167	−0.007
Ascorbic acid (X2, mg/100 g)	−0.590	−0.671	−0.283	0.176	−0.087
Fresh mass (X3, g g)	0.039	0.177	0.922	0.161	−0.128
Shape ratio (X4)	0.319	−0.068	0.123	0.792	0.023
Edible rate (X5, %)	0.114	0.345	0.800	−0.166	0.110
Moisture content (X6, %)	−0.848	−0.332	−0.085	−0.303	−0.067
Na (X7, mg/kg)	0.842	0.120	−0.021	−0.164	0.226
Mg (X8, mg/kg)	0.915	0.069	−0.152	0.266	0.049
K (X9, mg/kg)	0.932	0.182	0.079	0.228	−0.015
Mn (X10, mg/kg)	0.470	0.230	0.034	0.597	0.304
Fe (X11, mg/kg)	0.684	0.376	−0.148	0.327	0.280
Cu (X12, mg/kg)	0.399	0.030	−0.718	−0.020	0.062
Zn (X13, mg/kg)	0.057	−0.105	−0.168	0.139	0.919
Dietary fiber (X14, %)	0.525	0.388	0.327	−0.204	0.462
cAMP (X15, mg/kg)	0.453	0.317	0.868	0.343	−0.136
Aspartate (X16, g/100 g)	0.568	0.255	0.474	0.360	−0.007
Threonine (X17, g/100 g)	0.710	0.426	0.280	0.392	0.066
Serine (X18, g/100 g)	0.659	0.433	0.231	0.259	−0.021
Glutamate (X19, g/100 g)	0.694	0.638	−0.100	0.162	0.075
Proline (X20, g/100 g)	0.658	0.362	−0.127	0.168	−0.251
Glycine (X21, g/100 g)	0.608	0.592	0.280	0.213	−0.020
Alanine (X22, g/100 g)	0.554	0.691	0.258	0.031	0.011
Valine (X23, g/100 g)	0.641	0.646	0.286	0.093	0.077
Isoleucine (X24, g/100 g)	0.561	0.695	−0.032	0.228	−0.271
Leucine (X25, g/100 g)	0.563	0.737	0.116	0.190	−0.048
Tyrosine (X26, g/100 g)	0.092	0.736	0.121	−0.348	−0.085
Phenylalanine (X27, g/100 g)	0.406	0.779	0.182	0.156	−0.066
Lysine (X28, g/100 g)	0.329	0.738	0.277	0.295	−0.120
Histidine (X29, g/100 g)	0.404	0.704	0.289	−0.056	0.196
Arginine (X30, g/100 g)	−0.326	0.749	0.081	0.119	0.455
Total amino acid (X31, mg/100 g)	0.794	0.518	0.116	0.222	−0.152

**Table 4:** (**a**) Classification of Chinese jujube samples based on eight main quality parameters by the OPLS-DA model; (**b**) discriminating accuracy of main quality parameters in Chinese jujube samples using the OPLS-DA model.

**(a)**												
**Data set**	**Region**	**Sample Number**	**Discriminant Accuracy (%)**	**Henan**			**Hebei**			**Shanxi**	**Shaanxi**	**Xinjiang**
**Training set**	Henan	11	100	11			0			0	0	0
	Hebei	11	100	0			11			0	0	0
	Shanxi	11	100	0			0			11	0	0
	Shaanxi	20	100	0			0			0	20	0
	Xinjiang	28	100	0			0			0	0	28
**Testing set**	Henan	4	100	4			0			0	2	0
	Hebei	4	75	0			3			0	1	0
	Shanxi	4	100	0			0			4	0	0
	Shaanxi	7	71.43	0			2			0	5	0
	Xinjiang	11	100	0			0			0	0	11
**(b)**												
**Data set**	**Region**	**Sample Number**	**Discriminant Accuracy (%)**	**Huizao**		**Jinsixiaozao**		**Hupingzao**		**Xiangzao**	**Tanzao**	**Junzao**
**Training set**	Huizao	24	100	11		0		0		0	0	0
	Jinsixiaozao	11	100	0		11		0		0	0	0
	Hupingzao	11	100	0		0		11		0	0	0
	Xiangzao	9	100	0		0		0		11	0	0
	Tanzao	11	100	0		0		0		0	11	0
	Junzao	15	100	0		0		0		0	0	15
**Testing set**	Huizao	9	100	9		0		0		0	0	0
	Jinsixiaozao	4	100	0		4		0		0	0	0
	Hupingzao	4	75	0		0		3		1	0	0
	Xiangzao	3	100	0		0		0		3	0	0
	Tanzao	4	75	0		1		0		0	3	0
	Junzao	6	100	0		0		0		0	0	6

## Data Availability

Not applicable.

## Supplementary Materials

The following are available online at https://www.mdpi.com/article/10.3390/foods10102270/s1, Figure S1: Correlations among 31 quality indicators of Chinese jujubes; Table S1: Reference conditions of sample digester; Table S2: Contents of 15 individual amino acids in Chinese jujubes from different categories; Table S3: Characteristics of 31 quality indicators of Chinese jujubes; Table S4: The scores and ranking of Chinese jujubes of different categories.

## References

[1] Liu X.X., Liu H.M., Yan Y.Y., Fan L.Y., Yang J.N., Wang X.D., Qin G.Y. (2020). Structural characterization and antioxidant activity of polysaccharides extracted from jujube using subcritical water. LWT.

[2] Sheng J.P., Shen L., Yahia E.M. (2011). Chinese jujube (*Ziziphus jujuba* Mill.) and Indian jujube (*Ziziphus mauritiana* Lam.). Postharvest Biology and Technology of Tropical and Subtropical Fruits.

[3] Tang Q., Li C., Ge Y., Li X., Cheng Y., Hou J., Li J. (2020). Exogenous application of melatonin maintains storage quality of jujubes by enhancing anti-oxidative ability and suppressing the activity of cell wall-degrading enzymes. LWT.

[4] Lin X., Zhou L., Li T., Brennan C., Fu X., Liu R.H. (2017). Phenolic content, antioxidant and antiproliferative activities of six varieties of white sesame seeds (*Sesamum indicum* L.). RSC Adv..

[5] Pon Y.L., Auersperg N., Wong A.S. (2005). Gonadotropins regulate N-cadherin-mediated human ovarian surface epithelial cell survival at both post-translational and transcriptional levels through a cyclic AMP/protein kinase A pathway. J. Biol. Chem..

[6] Chen K., Fan D., Fu B., Zhou J., Li H. (2019). Comparison of physical and chemical composition of three chinese jujube (*Ziziphusjujuba* Mill.) cultivars cultivated in four districts of Xinjiang region in China. Food Sci. Technol..

[7] Song J., Chen Q., Bi J., Meng X., Wu X., Qiao Y., Lyu Y. (2020). GC/MS coupled with MOS e-nose and flash GC e-nose for volatile characterization of Chinese jujubes as affected by different drying methods. Food Chem..

[8] Gao Q.H., Wu P.T., Liu J.R., Wu C.S., Parry J.W., Wang M. (2011). Physico-chemical properties and antioxidant capacity of different jujube (*Ziziphus jujuba* Mill.) cultivars grown in loess plateau of China. Sci. Hortic..

[9] Chen T., Zhao Y., Zhang W., Yang S., Ye Z., Zhang G. (2016). Variation of the light stable isotopes in the superior and inferior grains of rice (*Oryzasativa* L.) with different geographical origins. Food Chem..

[10] Qian J., Dai B., Wang B., Zha Y., Song Q. (2020). Traceability in food processing: Problems, methods, and performance evaluations-a review. Crit. Rev. Food Sci. Nutr..

[11] Gonzalez-Dominguez R., Sayago A., Akhatou I., Fernandez-Recamales A. (2020). Multi-chemical profiling of strawberry as a traceability tool to investigate the effect of cultivar and cultivation conditions. Foods.

[12] Zhang T., Wang Q., Li J., Zhao S., Qie M., Wu X., Bai Y., Zhao Y. (2021). Study on the origin traceability of Tibet highland barley (*Hordeum vulgare* L.) based on its nutrients and mineral elements. Food Chem..

[13] Sun L., Ma X., Jin H.Y., Fan C.J., Li X.D., Zuo T.T., Ma S.C., Wang S. (2020). Geographical origin differentiation of Chinese Angelica by specific metal element fingerprinting and risk assessment. Environ. Sci. Pollut. Res. Int..

[14] Zhang Y.Y., Mao X.H., Zhang J.Y. (2018). Effects of different extraction methods on cAMP from wild jujube. Food Sci. Technol..

[15] Varra M.O., Ghidini S., Zanardi E., Badiani A., Ianieri A. (2019). Authentication of European sea bass according to production method and geographical origin by light stable isotope ratio and rare earth elements analyses combined with chemometrics. Ital. J. Food Saf..

[16] Tian Y., Gou X., Niu P., Sun L., Guo Y. (2018). Multivariate data analysis of the physicochemical and phenolic properties of not from concentrate apple luices to explore the alternative cultivars in juice production. Food Anal. Methods.

[17] Chen L., Yu F., Sun S., Liu X., Sun Z., Cao W., Liu S., Li Z., Xue C. (2021). Evaluation indicators of Ruditapes philippinarum nutritional quality. J. Food Sci.Technol..

[18] Xue Y.L., Chen J.N., Han H.T., Liu C.J., Gao Q., Li J.H., Li D.J., Tanokura M., Liu C.Q. (2019). Multivariate analyses of the physicochemical properties of turnip (*Brassicarapa* L.) chips dried using different methods. Dry. Technol..

[19] Shin E.C., Pegg R.B., Phillips R.D., Eitenmiller R.R. (2010). Interrelationships among tocopherols of commercial Runner market type peanuts grown in the United States. Int. J. Food Sci. Technol..

[20] Bi J.F., Wang X., Chen Q.Q., Liu X., Wu X.Y., Wang Q., Lv J., Yang A.J. (2015). Evaluation indicators of explosion puffing Fuji apple chips quality from different Chinese origins. LWT—Food Sci. Technol..

[21] Liu C.J., Wang H.O., Xue Y.L., Zhang Z.Y., Niu L.Y., Li D.J., Jiang N., Cui L., Liu C.Q. (2017). Screening quality evaluation factors of freezedried peach (*Prunus persica* L. Batsch) powders from different ripening time cultivars. J. Food Qual..

[22] He X., Yangming H., Gorska-Horczyczak E., Wierzbicka A., Jelen H.H. (2021). Rapid analysis of Baijiu volatile compounds fingerprint for their aroma and regional origin authenticity assessment. Food Chem..

[23] Cheaitou A., van Delft C., Dallery Y., Jemai Z. (2009). Two-period production planning and inventory control. Int. J. Prod. Econ..

[24] Jia C., Shan C., Zhou T., Xiangyang L.I., Peng W.U., Sun Y. (2019). Comparison of fruit nutritional traits of major cultivars of Chinese cherry (Prunus pseudocerasus Lindl.). Food Sci..

[25] Jiang T., He F., Han S., Chen C., Zhang Y., Che H. (2019). Characterization of cAMP as an anti-allergic functional factor in Chinese jujube (*Ziziphus jujuba* Mill.). J. Funct. Foods.

[26] Ibrahim S.A., Ayad A.A., Williams L.L., Ayivi R.D., Gyawali R., Krastanov A., Aljaloud S.O. (2020). Date fruit: A review of the chemical and nutritional compounds, functional effects and food application in nutrition bars for athletes. Int. J. Food Sci. Technol..

[27] Chang S.K., Alasalvar C., Shahidi F. (2016). Review of dried fruits: Phytochemicals, antioxidant efficacies, and health benefits. J. Funct. Foods.

[28] Hassan I., Cotrozzi L., Haiba N.S., Basahi J., Hammam E. (2017). Trace elements in the fruits of date palm (*Phoenix dactylifera* L.) in Jeddah City, Saudi Arabia. Agrochimica.

[29] Hasnaoui A., Elhoumaizi M.A., Asehraou A., Sindic M., Hakkou A. (2010). Chemical composition and microbial quality of dates grown in Figuig oasis of Marocco. Int. J. Agric. Biol..

[30] Guo S., Duan J.A., Qian D., Tang Y., Wu D., Su S., Wang H., Zhao Y. (2015). Content variations of triterpenic acid, nucleoside, nucleobase, and sugar in jujube (*Ziziphu sjujuba*) fruit during ripening. Food Chem..

[31] Gao Q.H., Wu C.S., Yu J.G., Wang M., Ma Y.J., Li C.L. (2012). Textural characteristic, antioxidant activity, sugar, organic acid, and phenolic profiles of 10 promising jujube (*Ziziphusjujuba* Mill.) selections. J. Food Sci..

[32] Zhang R., Sun X., Zhang K., Zhang Y., Song Y., Wang F. (2020). Fatty acid composition of 21 cultivars of Chinese jujube fruits (*Ziziphusjujuba* Mill.). J. Food Meas. Charact..

[33] Reche J., Almansa M.S., Hernandez F., Carbonell-Barrachina A.A., Legua P., Amoros A. (2019). Fatty acid profile of peel and pulp of Spanish jujube (*Ziziphusjujuba* Mill.) fruit. Food Chem..

[34] Rahimi A., Banakar A., Zareiforush H., Beygvand M., Montazeri M. (2014). Classification of Jujube fruits using different data mining methods. Researcher.

[35] Guo Y., Ni Y., Kokot S. (2016). Evaluation of chemical components and properties of the jujube fruit using near infrared spectroscopy and chemometrics. Spectrochim. Acta A Mol. Biomol. Spectrosc..

[36] Eriksson L., Trygg J., Wold S. (2008). CV-ANOVA for significance testing of PLS and OPLS^®^ models. J. Chemom..

