# Supplementary material for: Geographical and Varietal Traceability of Chinese Jujubes Based on Physical and Nutritional Characteristics
Source: Foods. 2021 Sep 25;10(10):2270. doi: 10.3390/foods10102270 (PMC8534552; doi:10.3390/foods10102270)
Supplement: Supplementary file 1 [file foods-10-02270-s001.zip › foods-1374448-supplementary.pdf]

Table S1 Reference conditions of sample digester

Step	Control temperature(℃)	Heating time (min)	incubation time(min)
1	120	5	5
2	150	5	10
3	190	5	20

Table S2 Contents of 15 individual amino acids in Chinese jujubes from different categories.

Category	aspartate (g/100g)	threonine (g/100g)	serine (g/100g)	glutamate (g/100g)	proline (g/100g)	glycine (g/100g)	alanine (g/100g)	valine (g/100g),	isoleucine (g/100g)	leucine (g/100g)	tyrosine (g/100g)	phenylalanine (g/100g)	lysine (g/100g)	histidine (g/100g)	arginine (g/100g)
Henan Huizao	0.23±0.04c	0.030±0.00c	0.034±0.01b	0.058±0.00d	0.52±0.04c	0.028±0.00e	0.026±0.01c	0.030±0.00c	0.026±0.005bc	0.048±0.005c	0.028±0.005b	0.042±0.005d	0.040±0.000c	0.010±0.000c	0.020±0.000d
Hebei Jinsixiaozao	0.24±0.04c	0.030±0.00c	0.030±0.00b	0.060±0.01d	0.40±0.04de	0.030±0.00de	0.030±0.00c	0.030±0.00c	0.020±0.000d	0.040±0.000d	0.020±0.000c	0.038±0.005d	0.038±0.004c	0.012±0.004c	0.020±0.000d
Shanxi Hupingzao	0.27±0.01bc	0.030±0.00c	0.036±0.01b	0.062±0.00d	0.33±0.03e	0.030±0.00de	0.036±0.01b	0.040±0.00b	0.022±0.004cd	0.048±0.005c	0.034±0.009ab	0.048±0.005c	0.042±0.004c	0.018±0.004b	0.040±0.007b
Shaanxi Xiangzao	0.25±0.14c	0.030±0.00c	0.038±0.01b	0.065±0.01cd	0.56±0.05c	0.035±0.01cd	0.040±0.00b	0.040±0.00b	0.030±0.000b	0.053±0.005c	0.040±0.000a	0.050±0.000bc	0.048±0.005b	0.020±0.000ab	0.025±0.006d
Shaanxi Tanzao	0.23±0.02c	0.034±0.01c	0.034±0.01b	0.074±0.01c	0.43±0.02d	0.038±0.00c	0.038±0.00b	0.040±0.00b	0.030±0.000b	0.060±0.000b	0.040±0.000a	0.050±0.000bc	0.050±0.000b	0.020±0.000ab	0.054±0.005a
Xinjiang Junzao	0.44±0.03a	0.053±0.01a	0.053±0.01a	0.087±0.01b	0.71±0.08b	0.050±0.00a	0.048±0.00a	0.055±0.01a	0.037±0.005a	0.070±0.000a	0.035±0.005a	0.057±0.005a	0.058±0.004a	0.025±0.005a	0.035±0.005bc
Region	0.33±0.07b	0.047±0.01b	0.050±0.01a	0.098±0.01a	0.85±0.12a	0.045±0.01b	0.047±0.01a	0.053±0.01a	0.037±0.005a	0.068±0.008a	0.038±0.004a	0.055±0.005ab	0.050±0.006b	0.023±0.005ab	0.032±0.004c

Table S3 Characteristics of 31qualityindicators of Chinese jujubes.

Quality parameter	Mean	Range	Standard deviation	CV (%)[a]
soluble sugar (%)	28.13	18.10-40.40	7.92	28.16
ascorbic acid (mg/100g)	171.22	19.00-357.00	113.61	66.35
fresh mass(g)	13.68	5.49-31.25	6.89	50.37
shape ratio	1.37	0.73-1.75	0.17	12.69
edible rate(%)	94.02	91.71-96.41	1.27	1.35
moisture content(%)	57.41	31.70-71.50	12.97	22.59
Na(mg/kg)	100.53	2.90-519.70	135.65	134.93
Mg(mg/kg)	244.56	133.00-536.00	93.27	38.14
K(mg/kg)	4225.50	1918.00-9275.00	1954.22	46.25
Mn(mg/kg)	2.23	1.20-3.40	0.50	22.22
Fe(mg/kg)	7.64	2.80-21.00	4.80	62.86
Cu(mg/kg)	1.27	0.57-2.30	0.39	30.96
Zn(mg/kg)	2.94	1.60-4.20	0.71	23.98
dietary fiber(%)	6.08	4.51-6.88	0.69	11.39
cAMP (mg/kg)	87.87	22.66-246.43	63.15	71.86
aspartate(g/100g)	0.29	0.030-0.49	0.095	32.61
threonine (g/100g)	0.037	0.030-0.060	0.010	26.96
serine (g/100g)	0.040	0.030-0.060	0.010	26.05
glutamate(g/100g)	0.073	0.050-0.11	0.017	22.59
proline (g/100g)	0.56	0.29-0.97	0.19	34.03
glycine (g/100g)	0.037	0.020-0.050	0.0088	23.71
alanine (g/100g)	0.038	0.020-0.050	0.0088	22.92
valine (g/100g)	0.042	0.030-0.060	0.010	24.75
isoleucine(g/100g)	0.029	0.020-0.040	0.0073	25.07
leucine(g/100g)	0.056	0.040-0.080	0.012	20.55
tyrosine(g/100g)	0.034	0.020-0.040	0.0080	23.75
phenylalanine(g/100g)	0.049	0.030-0.060	0.0075	15.28
lysine(g/100g)	0.047	0.030-0.060	0.0079	16.76
histidine(g/100g)	0.019	0.010-0.030	0.0064	34.35
arginine(g/100g)	0.033	0.020-0.060	0.012	37.05
total amino acid (mg/100g)	1.38	0.97-2.07	0.34	24.74

b "CV" represents "coefficient of variation"

Table S4 The scores and ranking of Chinese jujubes of different categories.

categories	PC score					Synthesis score	Ranking
	**PC1**	**PC 2**	**PC 3**	**PC 4**	**PC 5**		
Henan Huizao1	-3.43	-1.78	-1.10	-1.01	0.95	-2.64	28
Henan Huizao2	-4.83	-2.87	-1.72	-0.08	0.91	-3.72	36
Henan Huizao3	-3.64	-1.92	-1.16	-0.97	1.21	-2.79	29
Henan Huizao4	-4.44	-1.68	-1.25	-1.17	0.97	-3.32	32
Henan Huizao5	-3.02	-0.79	-1.54	-2.35	1.23	-2.34	27
Hebei Jinsixiaozao1	-4.99	-1.90	-1.01	0.80	-1.46	-3.68	35
Hebei Jinsixiaozao2	-4.65	-0.50	-1.10	-0.10	-0.98	-3.30	31
Hebei Jinsixiaozao3	-4.95	-1.92	-0.39	0.59	-0.17	-3.56	34
Hebei Jinsixiaozao4	-3.97	-1.47	-0.31	1.21	-0.75	-2.83	30
Hebei Jinsixiaozao5	-4.67	-1.95	-0.45	1.27	-0.58	-3.36	33
Shanxi Hupingzao1	-2.17	1.06	-0.48	2.89	0.12	-1.14	23
Shanxi Hupingzao2	-2.12	2.14	0.52	0.97	-0.63	-1.03	22
Shanxi Hupingzao3	-2.83	2.05	0.76	1.37	-0.70	-1.48	25
Shanxi Hupingzao4	-2.98	1.88	-0.58	-0.29	-1.20	-1.85	26
Shanxi Hupingzao5	-1.81	2.07	0.56	-0.03	-0.38	-0.89	21
Shaanxi Xiangzao1	-1.81	1.32	0.57	-2.19	-1.25	-1.17	24
Shaanxi Xiangzao2	-1.47	1.19	1.47	-2.42	-0.38	-0.86	20
Shaanxi Xiangzao3	-1.07	1.17	0.89	-1.59	-0.28	-0.59	17
Shaanxi Xiangzao4	-1.30	2.21	0.51	-2.04	-0.49	-0.67	18
Shaanxi Tanzao1	-1.00	1.04	2.13	1.27	1.92	-0.17	14
Shaanxi Tanzao2	-1.15	0.39	2.42	2.12	2.03	-0.27	15
Shaanxi Tanzao3	-0.81	0.83	1.44	1.08	2.16	-0.13	13
Shaanxi Tanzao4	-1.18	2.06	1.08	1.00	1.27	-0.28	16
Shaanxi Tanzao5	-1.78	2.61	2.76	-1.05	-0.35	-0.67	19
Xinjiang Junzao1	5.53	1.28	-1.90	-0.54	0.87	3.70	7
Xinjiang Junzao2	5.01	1.57	-1.74	-1.23	0.95	3.37	10
Xinjiang Junzao3	7.14	0.58	-2.18	0.27	1.23	4.72	1
Xinjiang Junzao4	6.25	2.22	-1.77	-0.45	-0.02	4.29	2
Xinjiang Junzao5	5.16	1.60	-2.17	1.16	-0.42	3.53	9
Xinjiang Junzao6	5.33	2.03	-2.33	1.79	-1.22	3.69	8
Xinjiang Huizao7	7.01	-4.43	0.74	1.08	0.05	4.18	4
Xinjiang Huizao1	6.93	-2.70	1.02	-0.87	0.49	4.28	3
Xinjiang Huizao2	2.83	-1.27	0.82	0.33	-1.91	1.71	11
Xinjiang Huizao3	2.63	-1.52	0.63	0.59	-2.52	1.51	12
Xinjiang Huizao4	6.00	-2.08	2.66	-0.53	-0.24	3.88	6
Xinjiang Huizao5	6.24	-2.53	2.20	-0.90	-0.40	3.90	5

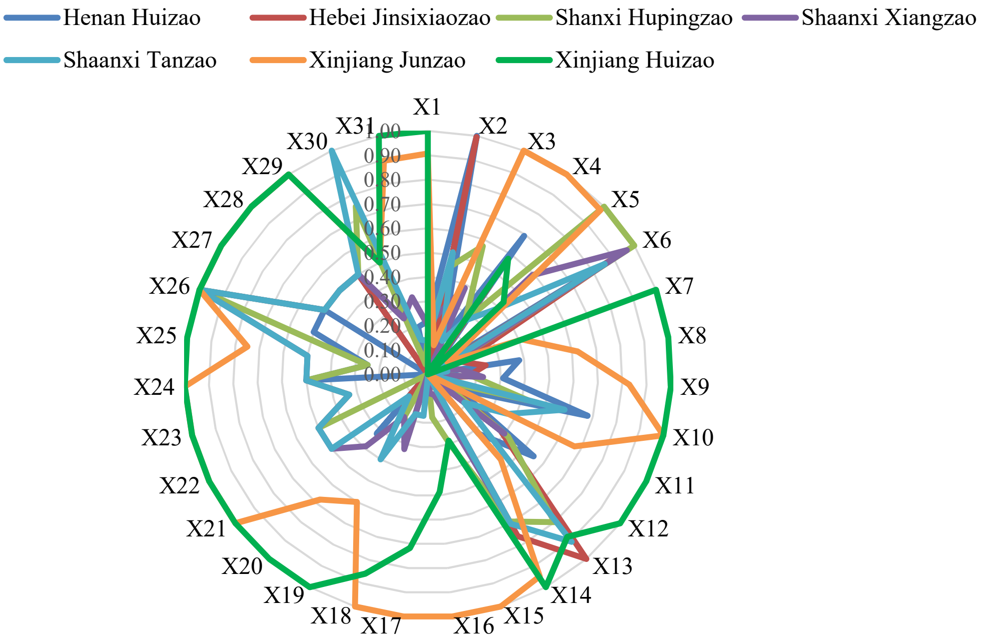

Figure S1. Correlations among 31 quality indicators of Chinese jujubes.

X1-X31: soluble sugar (%),ascorbic acid (mg/100g), fresh mass(g), shape ratio, edible rate (%), moisture content (%), Na (mg/kg), Mg (mg/kg), K (mg/kg), Mn (mg/kg), Fe (mg/kg), Cu(mg/kg), Zn(mg/kg), dietary fiber(%), cAMP (mg/kg), aspartate(g/100g), threonine (g/100g), serine (g/100g), glutamate (g/100g), proline (g/100g), glycine (g/100g), alanine (g/100g), valine (g/100g), isoleucine (g/100g), leucine (g/100g), tyrosine (g/100g), phenylalanine (g/100g), lysine (g/100g), histidine (g/100g), arginine (g/100g), total amino acid (mg/100g)
